# An interpretable machine learning approach to identify mechanism of action of antibiotics

**DOI:** 10.1038/s41598-022-14229-3

**Published:** 2022-06-20

**Authors:** Mihir Mongia, Mustafa Guler, Hosein Mohimani

**Affiliations:** Computational Biology Department, School of Computer Science, Carnegie Mellon University, Pittsburgh, USA

**Keywords:** Computational models, Data mining, Virtual drug screening

## Abstract

As antibiotic resistance is becoming a major public health problem worldwide, one of the approaches for novel antibiotic discovery is re-purposing drugs available on the market for treating antibiotic resistant bacteria. The main economic advantage of this approach is that since these drugs have already passed all the safety tests, it vastly reduces the overall cost of clinical trials. Recently, several machine learning approaches have been developed for predicting promising antibiotics by training on bioactivity data collected on a set of small molecules. However, these methods report hundreds/thousands of bioactive molecules, and it remains unclear which of these molecules possess a novel mechanism of action. While the cost of high-throughput bioactivity testing has dropped dramatically in recent years, determining the mechanism of action of small molecules remains a costly and time-consuming step, and therefore computational methods for prioritizing molecules with novel mechanisms of action are needed. The existing approaches for predicting bioactivity of small molecules are based on uninterpretable machine learning, and therefore are not capable of determining known mechanism of action of small molecules and prioritizing novel mechanisms. We introduce InterPred, an interpretable technique for predicting bioactivity of small molecules and their mechanism of action. InterPred has the same accuracy as the state of the art in bioactivity prediction, and it enables assigning chemical moieties that are responsible for bioactivity. After analyzing bioactivity data of several thousand molecules against bacterial and fungal pathogens available from Community for Open Antimicrobial Drug Discovery and a US Food and Drug Association-approved drug library, InterPred identified five known links between moieties and mechanism of action.

## Introduction

Antibiotic resistance bacteria kills 700,000 worldwide each year^1^. Overcoming the challenge of antibiotic resistance requires development of antibiotics that can kill bacteria using novel modes of action. While the need for novel antibiotics has become more urgent in recent years, the progress in development of novel therapeutics has slowed down due to hurdles in discovery pipelines and lack of economic incentives. With current trends it is forecast that by 2050, the mortality rate of antibiotic resistance bacteria will be over ten million worldwide^2^, surpassing that of cancer.

With hundreds of millions of known molecular structures available in molecular libraries^3–5^, methods for prediction of bioactivity solely based on chemical structure can aid in selecting promising molecules active against targets of interest for downstream bioactivity testing. The first techniques for predicting the relationship between chemical structure and activity were rule based^6^. The first machine learning approaches for prediction of structure-activity relationships from training data appeared in the 1990s^7^.

Neural networks were among the earliest methods used for learning structure-activity relationship from training data^8^. However until very recently, neural networks (and many of other machine learning methods) were not capable of directly taking the graph structure of molecules as input. Recent advances in graph-based machine learning have enabled representation of complex molecular structures with real-valued vectors, making it feasible to incorporate local/global structural information for the prediction of molecular properties^9^.

Recently, Stokes et al.^10^ applied a directed message passing neural network (D-MPNN) for prediction of the antibiotic activity of small molecules. In this approach, a vector representation is learned for each atom, and the molecular property is predicted as a learned non-linear transformation of these representations^11,12^. Stokes et al. reported strong antimicrobial activity for Halicin, a drug chemical compound originally developed for treatment of diabetes^13^.

One of the main bottlenecks of the existing approaches is that they usually report hundreds/thousands of molecules, where the majority of them possess known mechanisms of action. Overcoming antibiotic resistant pathogens crucially depends on finding small molecules with novel mechanism of action, and currently determining the mechanism of action for small molecules remains an expensive and time-consuming effort. Therefore, it is crucial to develop computational methods for determining molecules with known mechanisms of action and prioritize molecules with novel mechanisms.

Mechanism of action of small molecules are usually linked to their bioactive moieties. One way to extract these moieties would be to find features of the molecule graph that correlate to bioactivity. Methods such as recursive feature elimination^14^, boruta^15^, and lasso^16^ have been developed for this purpose, but they are limited to cases where a feature set is available.

Another method to find bioactive moieties is to determine the portion of a molecular graph that a D-MPNN uses to make a prediction. Several heuristic approaches have been developed in order to interpret graph neural networks. One approach is to take the gradient of neural networks with respect to the atoms in the molecular graph^17–20^ and to attribute atoms with more importance if the gradient value for an atom is large. The set of atoms determined to be important by this approach, however, are not necessarily biologically relevant as often a large portion of the molecular graph is flagged as important. Furthermore although gradient methods have had some empirical success, the gradient only represents how the model changes with small perturbations, and high gradient values for atoms do not necessarily mean those atoms are important for classification by a neural network. Another approach for interpreting graph neural networks is to exhaustively search all subgraphs of a molecular graph and find those subgraphs that are either subsets of important nodes as determined by the gradient method or those that do not change the output of the neural network significantly^17,21^. These methods again often fail in capturing reasonable bioactive moieties as they highlight subgraphs that are common in the molecular space.

Interpreting which substructures are responsible for bioactivity is a challenging problem for the existing algorithms, as there are an exponential number of substructures of molecular graphs, and it is impossible to correctly infer which of these millions of substructures are responsible for activity from a few thousand training points. One way to overcome this issue is to limit the candidate substructures to those that are biologically important, including simple ring structures and functional groups^22,23^. This knowledge however has rarely been integrated in machine learning methods for drug discovery.

In this work we develop an interpretable machine learning model by first identifying the simple ring structures and functional groups in the training data and using them to create binary feature vectors for each molecule where zeros and ones indicate absence/presence of rings and functional groups. Using simple rings and structures as features is advantageous since it is easier to interpret the correlations between these features and mechanism of action in the downstream analysis. Then we train a logistic regression or extra trees model with balanced scoring on these features in order to create a low complexity model that accounts for imbalanced data. Our model achieves similar accuracy as the D-MPNN in Stokes et al. while being fully interpretable, and it clusters molecules based on their mechanism of action. Moreover, the method can associate a bioacive molecule with its bioactive moiety, providing a strategy for prioritizing molecules with novel mechanism of action. Application of our method to the Community for Open Antimicrobial Drug Discovery (CO-ADD) and a FDA-approved dataset of antibacterial and antifungal bioactivites of several thousand molecules assigned five known mechanism of actions to their moieties.

## Results

### Overview of InterPred

**Figure 1:**
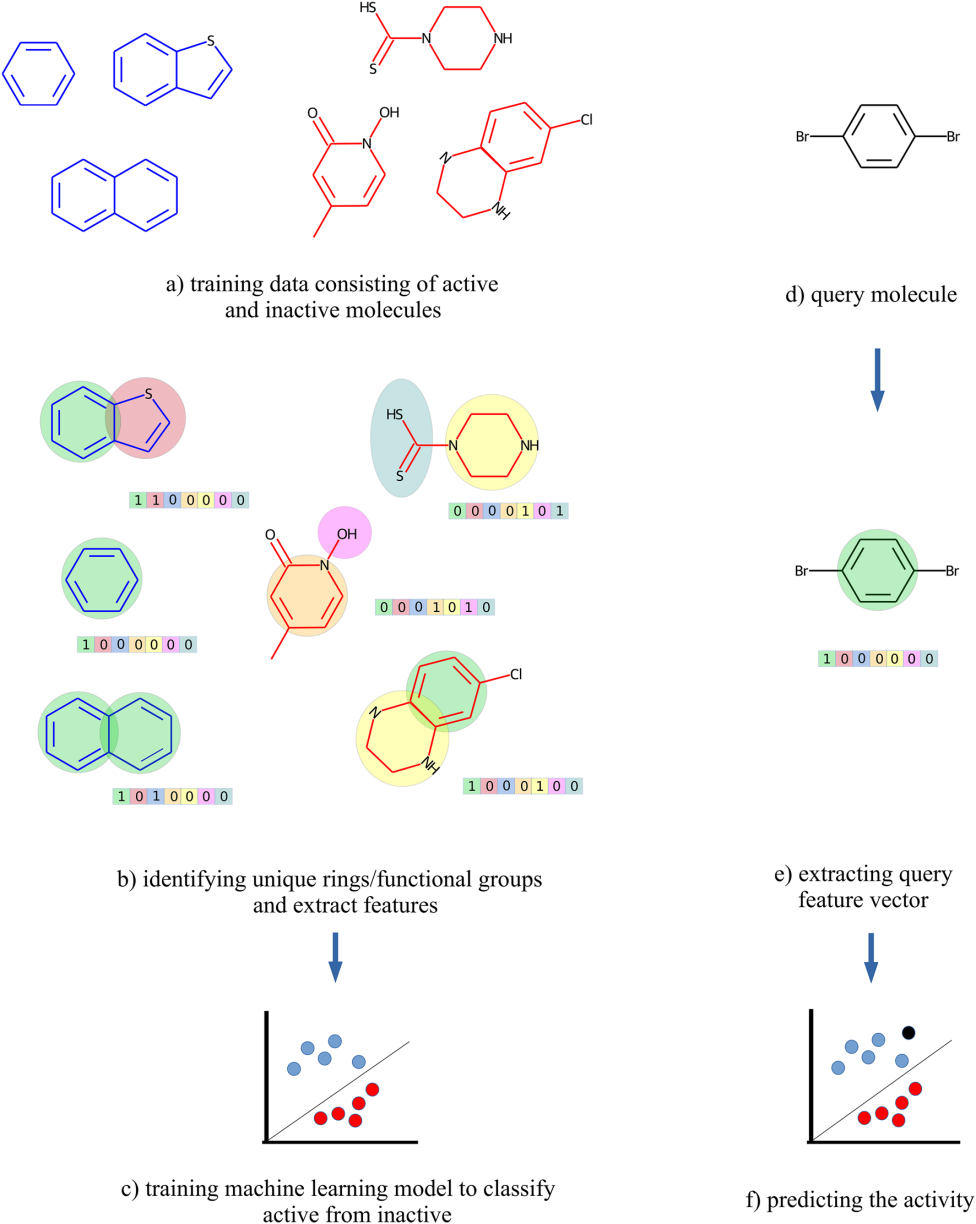
Interpretable prediction of antibiotic activity. Given (**a**) a collection of molecules, (**b**) InterPred finds all unique ring structures and functional groups and creates a binary vector for each molecule where zero/one indicates absence/presence of ring structure. Then (**c**) InterPred trains a logistic regression classifier with $l_1$-regularization and balanced scoring/extra trees classifier on the resulting binary features. Given (**d**) a query molecule, (**e**) InterPred extracts the binary features, and (**f**) applies the logistic regression classifier/extra trees classifier to predict the activity.

InterPred predicts bioactivity of small molecules in the following steps (Fig. 1). Given (a) a collection of molecules (b) all unique simple ring structures and functional groups are extracted into binary vectors where 0/1 indicates absence/presence of a substructure. Then, (c) extra trees/logistic regression classifier with $\ell _1$ regularization is trained on the extracted binary features using balanced scoring. Given (d) a query molecule, (e) binary features are extracted, and (f) the trained model is used for predicting bioactivity.

### Overview of MOACluster

**Figure 2:**
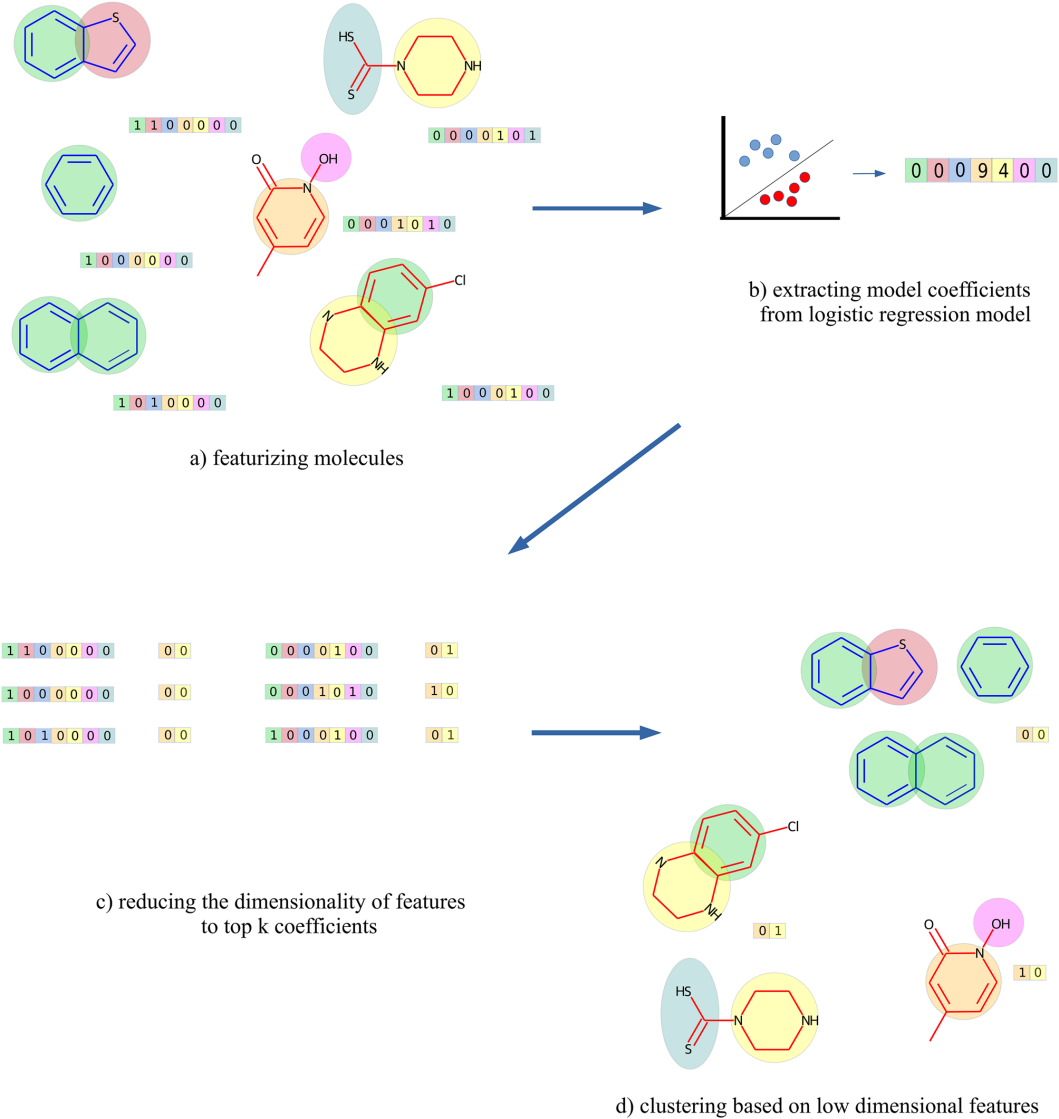
Clustering molecules based on their mechanism of action. Given (**a**) a collection of molecules, MOACluster extracts their binary features. Then (**b**) a logistic regression classifier is trained on the resulting binary features and the model coefficients are extracted. (**c**) MOACluster finds the indices of the top k coefficients and reduces molecule binary features to those k indices. (**d**) The molecules are clustered according to the reduced binary features.

MOACluster groups molecules with similar mechanism of action (MOA) in the following steps (Fig. 2). Given (a) a collection of molecules, MOACluster extracts their binary features. Then (b) a logistic regression classifier with $\ell _1$ regularization and balanced scoring is trained to predict bioactivity, and the model parameters are extracted. (c) MOACluster finds the indices of the top k coefficients and reduces molecule binary features to those k indices. (d) The molecules are clustered according to the reduced binary features.

### Datasets

InterPred was trained on two datasets. The first dataset contains molecules from a US Food and Drug (FDA)—approved library, along with 800 natural products isloated from plant, animal, and microbial sources (total of 2335 unique compounds)^10^. Data on growth inhibition against *Escheria coli* is available for all the molecules. The corresponding test data contains growth inhibition of 162 molecules from the Drug Repurposing Hub^22^. Each molecule in the test data is annotated with a mechanism of action by which it fights the disease it was originally purposed for. The second data set, CO-ADD^23,24^, contains bio-activity data from 4,803 molecules against seven bacterial and fungal pathogens, which include *Staphylococcus aureus*, *Pseudomonas aeruginosa*, *Acinetobacter baumannii*, *Candida albicans*, *Klebsiella pneumoniae*, *Cryptococcus neoformans*, and *Escheria coli* . For this dataset, 80% of the molecules are randomly selected for training and the rest are allocated for testing.

### Bioactivity prediction

**Figure 3:**
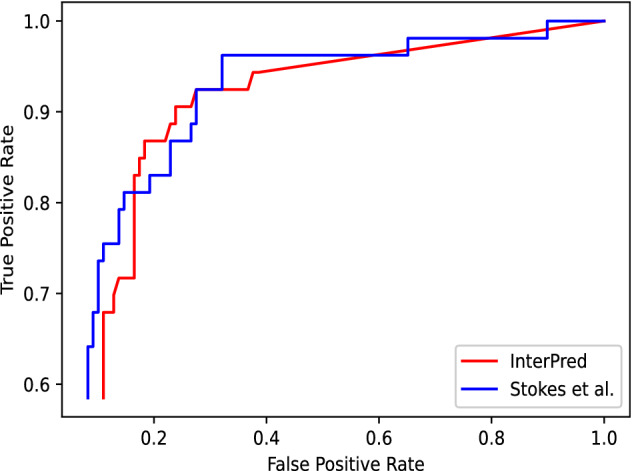
ROC curve for neural network model from Stokes et al. and InterPred. For false positive rates greater than 0.3, the models have nearly identical true positive rate.

**Figure 4:**
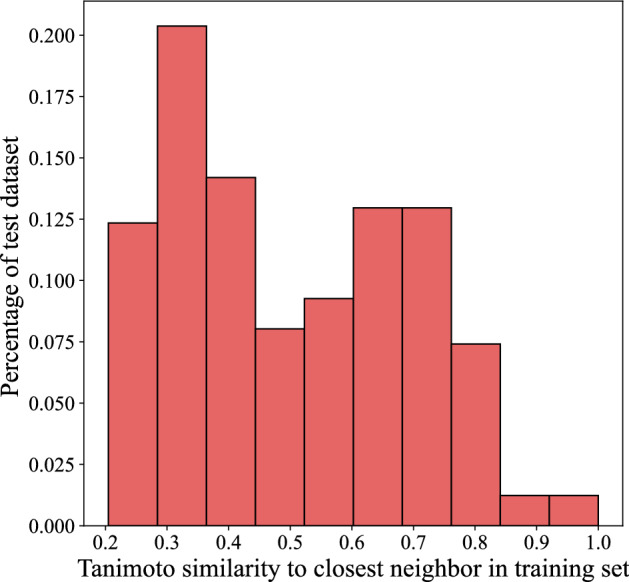
Distribution of the tanimoto similarity between each test data point and its nearest neighbor in the training dataset. The average tanimoto similarity between test data points and their closest neighbors is 0.5035 and the standard deviation is 0.18. Only 1.2% of test data points are more than 90% similar to a training data point.

Figure 3 illustrates the receiver operating characteristic (ROC) curve of InterPred compared to the approach from Stokes et al. on predicting activity against *E. coli*. Here 2335 molecules have been used for training, 162 molecules have been used for testing. These test molecules correspond to the portion of the Drug Repurposing Hub for which screening data against *E. coli* is available (Stokes et al.). Figure 4 shows the distribution of the tanimoto similarity between each test data point and their closest neighbor in the training dataset. InterPred achieves nearly the same accuracy as Stokes et al. The area under the curve (AUC) for InterPred is 0.87 while the AUC for Stokes et al. is 0.88. Unlike Stokes et al., InterPred uses fully interpretable features.

### Linking substructures to mechanism of action in Stokes et al. dataset

**Figure 5:**
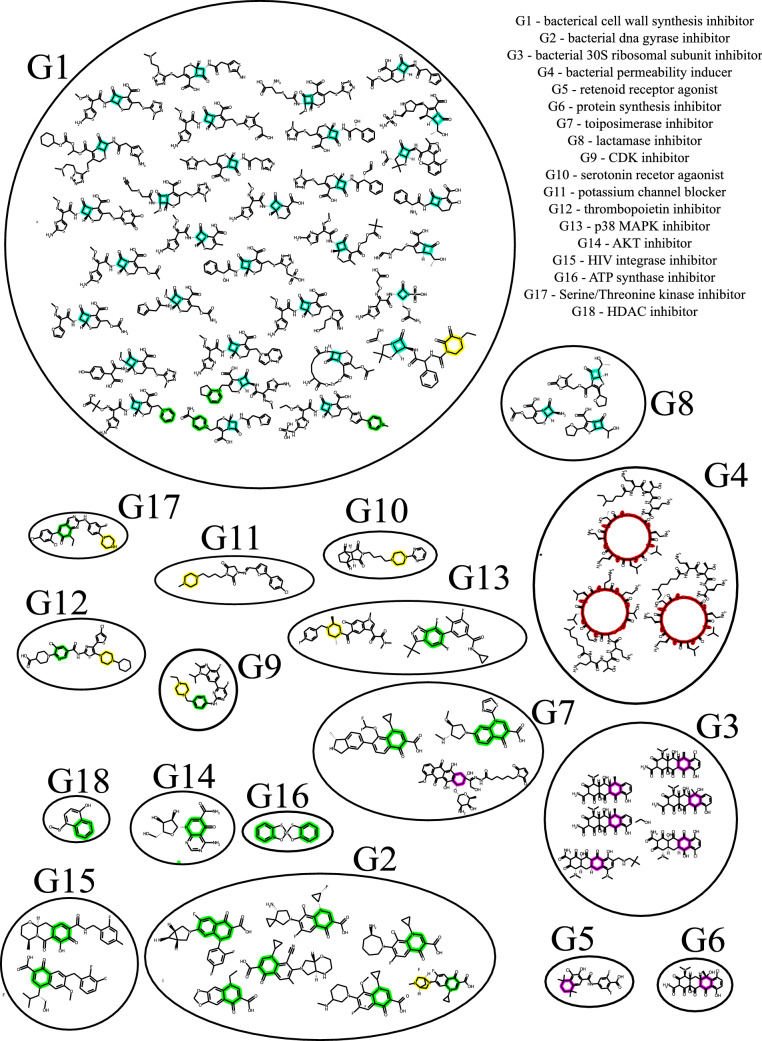
Mechanism of action of molecules containing at least one of the five most important simple rings according to the logistic regression model. Each of the five rings are highlighted with a different color. Molecules sharing the same mechanism of action, as reported in the Drug Repurposing Hub, are further circled together. For the majority of molecules with similar bioactive moiety, the mode of action is the same.

Figure 5 shows the mechanism of action of molecules containing at least one of the five most important simple rings according to the logistic regression model. For the majority of molecules with similar bioactive moiety, the mode of action is the same. For example beta-lactam rings (shown in blue), are present in antibiotics such as penicillins and cephalosporins, and they have been reported to prevent cell wall synthesis^25,26^. The majority of the molecules with this ring are mapped to the cell wall inhibition (G1) mechanism of action. In cases when molecules with the same moiety are mapped to multiple mechanisms of action, those mechanisms of action are usually similar. For example, for cyclohexane (shown in purple) associated mechanisms of action are bacterial 30S ribosomal subunit inhibitor (G3) and protein synthesis inhibitor (G6), both related to inhibiting protein synthesis. For moiety 4-quinolone (shown in green), the associated modes of action are HDAC inhibitor (G18), DNA gyrase inhibitor (G2), and topoisomerase inhibitor (G7), which are all related to inhibition of bacterial nucleic acid synthesis. Molecules containing 4-quinolone are known to inhibit bacterial nucleic acid synthesis by disrupting the enzymes topoisomerase IV and DNA gyrase^27^. In cases where two molecules contain distinct bioactive moieties, they usually have distinct mechanisms of action. The only exceptions G13 and G17 can be explained by the fact that MAP kinases are a subset of Serine/Threonine Kinases^28^. Among all the pairs of molecules with the same mechanism of action, 76% are clustered together by MOACluster, and among all the pairs of molecules clustered together by MOACluster, 67.6% have identical and 71% have similar mechanisms of action.

### Linking substructures to mechanism of action in CO-ADD dataset

**Figure 6:**
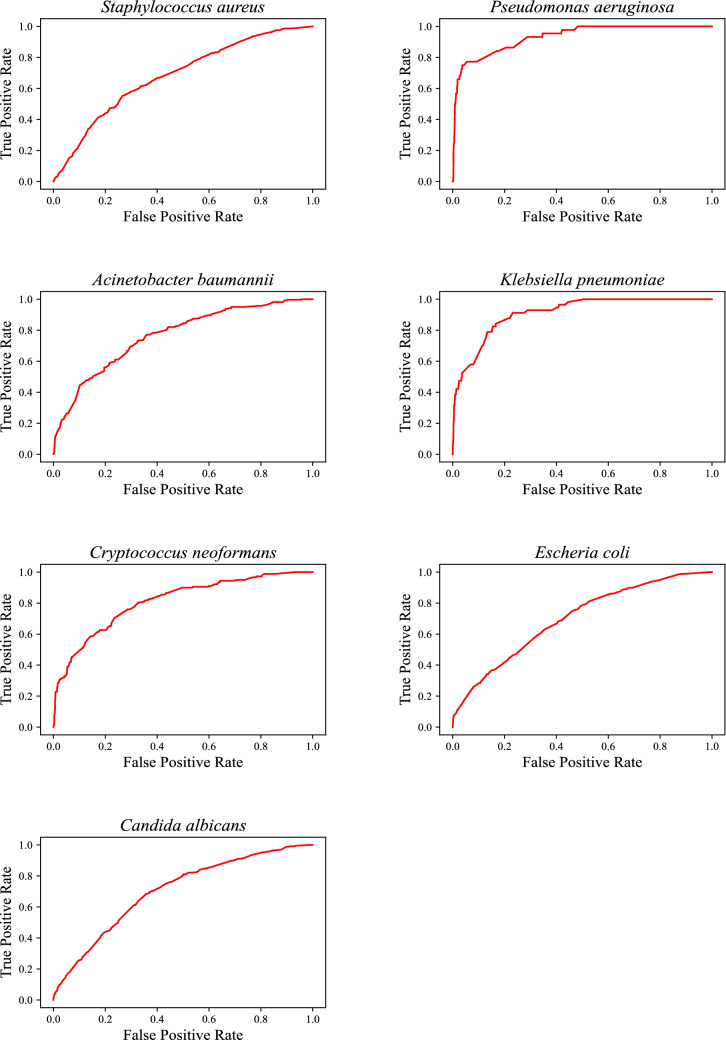
ROC curve of InterPred for prediction of growth inhibition for 7 different bacteria in the CO-ADD dataset.

**Figure 7:**
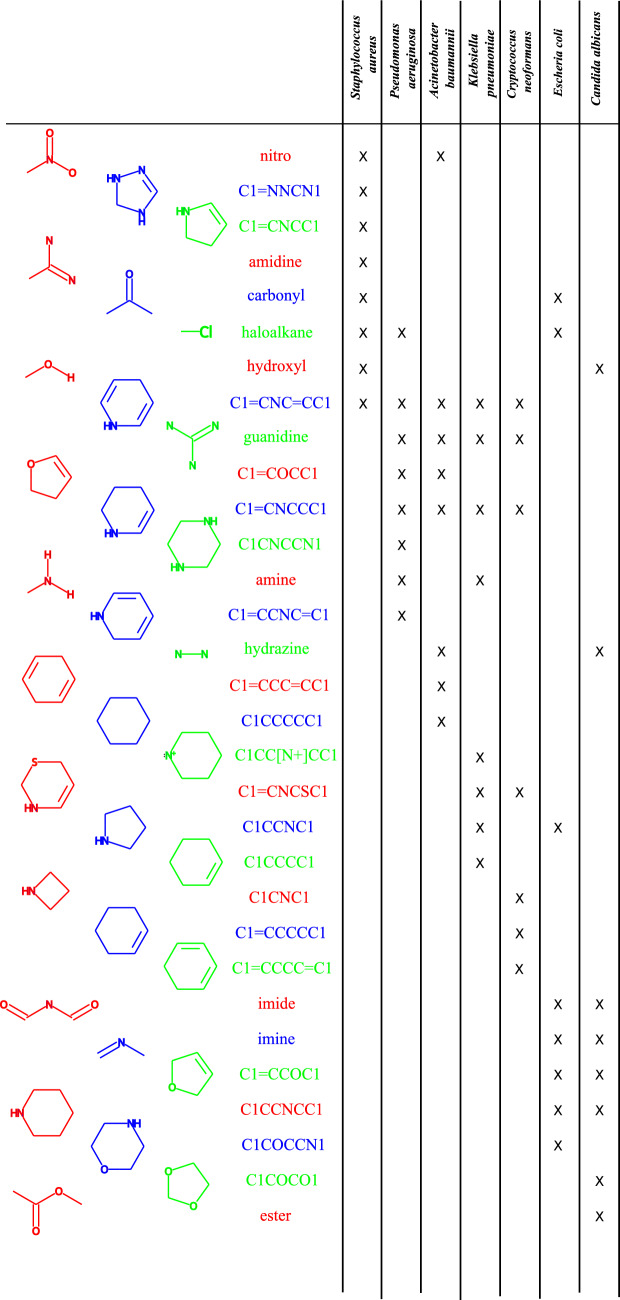
Top 31 ring/functional group features predicted to govern the mechanism of action of molecules along with pathogens they inhibit.

Figure 6 shows the ROC curve of InterPred for prediction for growth inhibition for seven different pathogens in the CO-ADD dataset^23,24^. Figure 7 shows the most dominant bio-active moieties detected by InterPred. The pathogens that are predicted to be inhibited by each moiety are also shown. It has been reported that guanadine^29–31^ and nitro^32^ are the bioactive moiety in various antibacterial molecules. Moreover hydrazone/hydarazine have been reported to be potent against *S. aureus*, *A. baumannii*, and *C. albicans*^33,34^.

## Discussion

Fighting antibiotic resistance requires discovery of small molecules with novel mechanism of action. Recent advances in high-throughput screening have made it possible to collect large databases of small molecules and their activities against resistant pathogens. Moreover, neural network models have been developed to predict the antimicrobial activity of molecules by training on these large datasets. However, the existing models are largely uninterpretable, and do not provide any insight about the mechanism of action of small molecules, and their bioactive moieties. Therefore, it remains unclear which of the molecules among their predictions has novel activity, and should be pushed toward pre-clinical trials.

One of the reasons graph neural network methods remain uninterpretable is that there are millions of molecular substructures and it is impossible to correctly identify which substructures are correlated to bioactivity when there are only a few thousand training data. By focusing on simple ring structures and other substructures that are known to be biologically important, InterPred bypasses this issue. When trained on bioactivity data of several thousand molecules from an FDA-approved library against *E. coli*, InterPred identified five important ring structures, each mapping to a distinct set of modes of action.

As massive public datasets of small molecule bioactivities are becoming available, it has become impossible to investigate all the molecules in these datasets that show interesting activities. Therefore, methods for mining these datasets and prioritizing for molecules with unseen mechanisms of actions are highly desired. InterPred is a fully interpretable approach for predicting the bioactivity of small molecules from training data and retains the same accuracy as the state of the art neural network approach. Additionally, InterPred finds chemical moieties in small molecule datasets that are responsible for bioactivity. Since molecules with novel bioactive moieties usually possess novel modes of action, if a molecule contains a previously unreported moiety that InterPred determines to be bioactive, the molecule could be prioritized for follow up studies.

## Methods

### Outline of InterPred algorithm

InterPred is an interpretable machine learning algorithm for prediction of bioactivity, functional groups responsible for bioactivity, and mechanism of action by training on data. Below we describe various steps of the InterPred algorithm.

### Extracting molecular features

Presence of simple rings are extracted using open source package rdkits^35^ by finding symmetrized smallest set of smallest rings. Additionally the presence 32 functional groups are extracted by checking whether each molecule has a graph substructure matching the functional group using the descriptors module in RDKit. These substructures are deduplicated using kekulized canonical SMILES^36^. Since small molecules have only a few simple rings, feature vectors for each molecules usually only have a few non-zero entries.

### Training

Both the extra trees ensemble classifier and logistic regression model with $\ell _{1}$ norm regularization are trained on training data and hyperparameters are optimized via five-fold cross validation. The number of trees in the extra trees model was cross-validated for the numbers 10, 40, 70, 100, 130, and 160. The lambda parameter for $\ell _1$-regularized logistic regression was cross-validated for values $\frac{1}{.01}$, $\frac{1}{.05}$, $\frac{1}{.1}$, $\frac{1}{.15}$, and $\frac{1}{.2}$. In logistic regression and extra trees, the loss function is of the form

1 $$\begin{aligned} {\text {min}} \ \ \ \sum _{t =1}^{T} L(f(\mathbf{x} ^{t}), y^{t}) \end{aligned}$$

where *t* is used as an index for each training point, $y^{t}$ represents the true label of each molecule in the training dataset, $\mathbf{x} ^{t}$ represents the features of each molecule, *f* is a function with range [0,1], and *L* refers to a loss function that is low when $f(x^{t})$ is close to $y^{t}$ and high otherwise. $y^{t}$ takes on value 1 if molecule *t* inhibits bacterial growth and 0 otherwise. In logistic regression, $f(\mathbf{x} ^{t}) = Sigmoid\big ((\mathbf{c} ^{T}x^{t})\big )$. $\mathbf{c}$ is the coefficient vector of logistic regression and $Sigmoid(z) = \frac{1}{1 + exp(-z)}$. $L(f(\mathbf{x} ^{t}), y^{t}) = CrossEntropy(f(\mathbf{x} ^{t}), y^{t}) + \lambda \frac{|\mathbf{c} |_{1}}{T}$ where $CrossEntropy(y,\hat{y}) = -ylog(\hat{y}) -(1 -y)log(1-\hat{y})$ and $\lambda$ is a regularization parameter optimized via cross validation. In extra trees $f(\mathbf{x} ^{t})$ is either 0 or 1 and is determined the by the majority label produced by all the trees in the extra trees ensemble. $L(f(\mathbf{x} ^{t}), y^{t})$ is 0 if $f(\mathbf{x} ^{t})$ and $y^{t}$ are not the same and 1 otherwise.

In the training set introduced by Stokes et al., nearly 95% of the molecules do not have antibacterial activity. Such an imbalance could result in misclassification of bioactive molecules as inactive. To avoid this, we use a “balanced” approach^37^. We modify the objective function in (1) to the following:

2 $$\begin{aligned} {\text {min}} \ \ \ \sum _{t =1}^{T} \frac{L(f(x^{t}), y^{t})}{b^{t}} \end{aligned}$$

where $b^{t}$ is the number of training points with label $y^{t}$. This way bioactive and inactive molecules will contribute to the training nearly equally.

### Identifying bioactive moeities

Substructures corresponding to largest positive coefficients of the logistic regression model are reported as bioactive moieties.

### Clustering mechanism of action

Since the logisitc regression model is trained with $\ell _1$ regularization, only a few coefficients are non-zero in the model. InterPred algorithm first reduces the feature vector for each molecule to these non-zero features, and then molecules with identical reduced feature vectors are assigned to the same cluster.

## Data Availability

The results present in the this study are available from https://gitlab.com/mongolicious/interpretableml-for-mechanism-of-action/. The datasets analyzed in Figs. 3, 4, and  5 are available at https://www.sciencedirect.com/science/article/pii/S0092867420301021. The datasets analyzed in Figs. 6 and 7 are available at https://db.co-add.org/.
